# Hyper-Activation of Notch3 Amplifies the Proliferative Potential of Rhabdomyosarcoma Cells

**DOI:** 10.1371/journal.pone.0096238

**Published:** 2014-05-05

**Authors:** Maria De Salvo, Lavinia Raimondi, Serena Vella, Laura Adesso, Roberta Ciarapica, Federica Verginelli, Antonio Pannuti, Arianna Citti, Renata Boldrini, Giuseppe M. Milano, Antonella Cacchione, Andrea Ferrari, Paola Collini, Angelo Rosolen, Gianni Bisogno, Rita Alaggio, Alessandro Inserra, Mattia Locatelli, Stefano Stifani, Isabella Screpanti, Lucio Miele, Franco Locatelli, Rossella Rota

**Affiliations:** 1 Department of Oncohematology, Ospedale Pediatrico Bambino Gesù, IRCCS, Roma, Italy; 2 Stanley Scott Cancer Center, Louisiana State University Health Sciences Center and Louisiana Cancer Research Consortium, New Orleans, Louisiana, United States of America; 3 Department of Pathology, Ospedale Pediatrico Bambino Gesù, IRCCS, Roma, Italy; 4 Pediatric Oncology Unit, Fondazione IRCCS Istituto Nazionale dei Tumori, Milano, Italy; 5 Anatomic Pathology Unit 2, Fondazione IRCCS Istituto Nazionale dei Tumori, Milano, Italy; 6 Department of Pediatrics, Oncohematology Unit, University of Padova, Padova, Italy; 7 Department of Pathology, University of Padova, Padova, Italy; 8 Department of Surgery, Ospedale Pediatrico Bambino Gesù, IRCCS, Roma, Italy; 9 Department of Scientific Directorate, Ospedale Pediatrico Bambino Gesù, IRCCS, Roma, Italy; 10 Centre for Neuronal Survival, Montreal Neurological Institute, McGill University, Montreal, Quebec, Canada; 11 Department of Molecular Medicine, Sapienza University, Roma, Italy; 12 Dipartimento di Scienze Pediatriche, Università di Pavia, Pavia, Italy; Cedars-Sinai Medical Center, United States of America

## Abstract

Rhabdomyosarcoma (RMS) is a pediatric myogenic-derived soft tissue sarcoma that includes two major histopathological subtypes: embryonal and alveolar. The majority of alveolar RMS expresses PAX3-FOXO1 fusion oncoprotein, associated with the worst prognosis. RMS cells show myogenic markers expression but are unable to terminally differentiate. The Notch signaling pathway is a master player during myogenesis, with Notch1 activation sustaining myoblast expansion and Notch3 activation inhibiting myoblast fusion and differentiation. Accordingly, Notch1 signaling is up-regulated and activated in embryonal RMS samples and supports the proliferation of tumor cells. However, it is unable to control their differentiation properties. We previously reported that Notch3 is activated in RMS cell lines, of both alveolar and embryonal subtype, and acts by inhibiting differentiation. Moreover, Notch3 depletion reduces PAX3-FOXO1 alveolar RMS tumor growth in vivo. However, whether Notch3 activation also sustains the proliferation of RMS cells remained unclear. To address this question, we forced the expression of the activated form of Notch3, Notch3IC, in the RH30 and RH41 PAX3-FOXO1-positive alveolar and in the RD embryonal RMS cell lines and studied the proliferation of these cells. We show that, in all three cell lines tested, Notch3IC over-expression stimulates in vitro cell proliferation and prevents the effects of pharmacological Notch inhibition. Furthermore, Notch3IC further increases RH30 cell growth in vivo. Interestingly, knockdown of Notch canonical ligands JAG1 or DLL1 in RMS cell lines decreases Notch3 activity and reduces cell proliferation. Finally, the expression of Notch3IC and its target gene HES1 correlates with that of the proliferative marker Ki67 in a small cohort of primary PAX-FOXO1 alveolar RMS samples. These results strongly suggest that high levels of Notch3 activation increase the proliferative potential of RMS cells.

## Introduction

Pediatric rhabdomyosarcoma (RMS) is a skeletal muscle-derived soft-tissue sarcoma affecting children and adolescents. It accounts for approximately 50% of all pediatric soft-tissue sarcomas and for 7–8% of all childhood malignancies [1]. Pediatric RMS includes two major histological subtypes, embryonal and alveolar [2]. Embryonal RMS has a favorable prognosis with survival rates of about 90% when nonmetastatic. Approximately 70% of alveolar RMSs harbor t(2;13) or t(1;13) chromosomal translocations that result in PAX3-FOXO1 or PAX7-FOXO1 oncoprotein expression. In particular, PAX3-FOXO1 may be a key biomarker patients' risk-stratification being correlated to the poorest outcome [3]. Despite improvement in multimodality treatments for high risk RMS, the management of those patients remains challenging, with a 5-year overall survival less than 30%. Therefore, understanding the molecular pathways that contribute to the pathogenesis and self-propagation of the most aggressive tumor forms is urgently needed.

RMS cells express key myogenic factors such as MyoD and Myogenin, but proliferate indefinitely and have lost the ability to terminally differentiate into skeletal myofibers [4].

The Notch signaling pathway plays fundamental roles in balancing proliferation versus differentiation [5] and is one of the major regulators of skeletal muscle tissue development. Mammals harbor four Notch genes, each encoding a type I trans-membrane Notch receptor paralog (Notch1–4). Notch receptors are most commonly activated after binding to the extracellular domain of a trans-membrane ligand of Delta-like (DLL1, DLL3–4) or Serrate/Jagged (JAG1–2) family on neighboring cells. The Notch-ligand interaction allows Notch to undergo sequential proteolytic cleavages, the last one being mediated by the γ-secretase complex that releases an active Notch intracellular domain (NotchIC). NotchIC translocates into the nucleus, where it behaves as a transcriptional regulator in complex with the DNA-binding RBP-Jk protein (also known as CSL/RBP-Jk, for CBF1/Su(H)/Lag1) inducing the expression of target genes [6]. Among canonical Notch target genes are those encoding the Enhancer of split group of transcriptional repressors, which are termed Hairy and Enhancer of split (HES) 1–7 and HES-related repressor (HEY) 1,2 and L in mammals [7].

In skeletal muscle progenitors, Notch1 activation impairs the transcription of myogenic regulatory factors, promoting proliferation and self-renewal of myogenic precursors [8], [9], [10], [11], [12]. Notch3 expression induces de-differentiation of myoblasts and, more recently, it has been shown to prevent myogenic differentiation by affecting Mef2c activity [13]. Consistent with these observations, inhibition of either γ-secretase activity or RBP-Jk-dependent gene transcription leads to myotube fusion [14], [15], [16].

Recently, we and others have shown that Notch signaling is deregulated in RMS [17], [18], [19], [20], [21]. General inhibition of Notch signaling with different approaches inhibits the proliferation of RMS cells [20] and prevents their migration and invasion [18]. Interestingly, the inhibition of the Notch1-HEY1 axis specifically impaired the proliferation of embryonal RMS cells, but it had only marginal effects on their differentiation properties [21]. Recently, we have shown that Notch3 prevented the differentiation of both subtypes of RMS cells [19]. Consistent with the data of Sang et al. [17], this function was, at least in part, related to the Notch3-dependent induction of HES1. We also reported that Notch3 inhibition hampered the growth of PAX3-FOXO1 alveolar RMS cells *in vivo* [19]. In addition, we observed that, in line with previous results [21], Notch1 knockdown was not sufficient to induce the differentiation of RMS cells, irrespective of their subtype [19]. These results are consistent with data showing that (i) myogenic differentiation and proliferation, though coupled, can be considered independent events during myogenesis [22] and (ii) different Notch paralogs can have different and even opposite roles in the same cellular/tissue context [23], [24], [25], [26], [27].

Therefore, we investigated the role of Notch3 activation by forcing the expression of an exogenous Notch3IC in RMS cells. We asked whether, in addition to regulating the differentiation of RMS cells, Notch3 may also play a role in sustaining their proliferation. Moreover, the relationship between Notch3 and/or HES1 expression and the proliferative status of primary RMS samples were evaluated.

We show here that Notch3IC over-expression in two PAX3-FOXO1-positive alveolar and one PAX3-FOXO1-negative embryonal RMS cell lines further increased their proliferative activity in vitro. Moreover, PAX3-FOXO1 alveolar cells over-expressing Notch3IC grew faster when xenografted into nude mice. Notably, forced over-expression of Notch3IC prevented the anti-proliferative effects of a γ-secretase inhibitor (GSI), suggesting that the inhibitory effect of this drug was mediated at least in part by Notch3 inhibition. We also expand our previous data showing that Notch3 signaling in RMS cells is not hyper-activated by a cell-autonomous mechanism, as reported in some other cancers [28], [29], [30], [31], but depends largely upon both types of canonical ligands. Finally, we report preliminary data showing that Notch3 activation and HES1 expression were highest in primary PAX-FOXO1-positive alveolar RMS samples with the highest levels of proliferative marker Ki67. Altogether, our results support a role for Notch3 activation as an amplifier of the proliferation potential of RMS cells and suggest that selected alveolar RMS may clinically benefit from either Notch inhibition or Notch ligand blockade

## Materials and Methods

### Tumor cell lines

RH30 (PAX3-FOXO1 expressing alveolar RMS) and RD (embrional RMS) cell lines were obtained from the American Type Culture Collection (ATCC, Rockville, MD, USA). RH41 (PAX3-FOXO1 expressing alveolar RMS) cell line was obtained from Deutsche Sammlung von Mikroorganismen und Zellkulturen GmbH (DSMZ, Braunschweig, Germany). RH30 and RH41 cells were cultured in RPMI 1640 (Invitrogen Corp., Carlsbad, CA, USA) while RD cells were cultured in DMEM (Invitrogen Corp., Carlsbad, CA, USA) supplemented with 10% FCS, 1% glutamine and 1% penicillin-streptomycin at 37°C in a humidified atmosphere of 5%CO2/95% air. Several first passage aliquots of each cell line were stored in liquid nitrogen at −80°C for subsequent assays. Each aliquot was passaged for a maximum of 3 months.

### Cell proliferation

For γ-secretase inhibitor treatment, 24 h after seeding (day0) in 6-well plates (5×104 cells/well) cells were treated with 5 µM of N-[N-(3,5-Difluoro-phenacetyl)-L-alanyl]-S-phenylglycine t-butyl Ester (DAPT) (Sigma, St Louis, MO, USA) in DMSO (10 mmol\L stock solution). Cells treated with DMSO alone (vehicle) were used as control. Cells were harvested at different time points and living cells were counted with the trypan blue exclusion method.

### Western blotting

Western blotting was performed on whole-cell lysates as previously described [32]. Antibodies against Notch1 (all forms; bTAN20) and Myogenin (F5D) were obtained from the Developmental Studies Hybridoma Bank at the University of Iowa (DSHB, Iowa City, IA, USA). Notch3 antibody (PAB-10683) was obtained from Orbigen Inc. (Orbigen, San Diego, CA, USA). Antibodies against Notch1-3 recognize an intracellular region within the γ-secretase cleavage product of each Notch molecule i.e., NotchIC. Antibodies against HES1 (sc-25392), p21Cip1 (sc-397), actin (sc-1616) and all secondary antibodies were obtained from Santa Cruz Biotechnology (Santa Cruz Biotechnology Inc., Santa Cruz, CA, USA). Antibodies against Phospho-Akt (Ser473), Akt (pan) (C67E7), Phospho-p38 (Thr180/Tyr182), p38 MAP Kinase, Phospho-p44/42 MAPK (ERK1/2) (Thr202/Tyr204), p44/42 MAPK (ERK1/2) (137F5), Jagged1 (28H8), DLL1 (2588) and GAPDH (D16H11) were obtained from Cell Signaling (Cell Signaling Technology Inc., Beverly, MA, USA). Antibody against tubulin (ab4074) was from Abcam (Abcam PLC., Cambridge, UK). All the antibodies were used in accordance with the manufacturer's instructions.

### Real time RT-PCR

Total RNA was extracted from RH30 cells using Trizol reagent (Invitrogen Corp., Carlsbad, CA, USA), according to the manufacturer's instructions. For the relative quantification of HES1 gene expression the TaqMan gene assay Hs00172878_m1 was used according to the manufacturer's instructions (Applied Biosystems, Life Technologies, Carlsbad, CA, USA).

### Plasmids and transfection

The plasmids pcDNA3.0\Notch3IC expressing the intracellular domain of human Notch3 (Notch3IC) (M. Bocchetta, Loyola University, Chicago, MC, USA) or the empty pcDNA3.0 were transfected using Lipofectamine 2000 (Invitrogen Corp., Carlsbad, CA, USA). Cells were selected with G418 (1.5 mg/ml) (4 weeks RH30 and RD and 1 week RH41 cells) and, then, used for the experiments.

### Soft-agar colony formation assay

Stably transfected Notch3IC or control vector RH30 cells were assayed for their capacity to form colonies in soft-agar as previously described [32]. Briefly, a total of 30000 cells was suspended in DMEM (10% FCS) containing 0.35% Noble agar (Sigma Chemical Co., St Louis, MO, USA). Cells were seeded on a layer of 0.7% Noble agar in DMEM (10% FCS) onto a 35-mm Petri dish. Medium was refreshed every 5 days. On week 4, the number of colonies per field was counted under the contrast-phase Eclipse TE200 microscope (Nikon, Sesto Fiorentino, Firenze, Italy). Two independent experiments were carried out in triplicate.

### Xenograft growth

Athymic 6-week-old female BALB/c nude mice (nu+\nu+) were purchased from Charles River. Procedures involving animals and their care conformed to institutional guidelines that comply with national and international laws and policies (EEC Council Directive 86\609, OJ L 358, 12 December 1987). The protocol was approved by the Committee on the Ethics of Animal Experiments of the Italian Ministry of Health to the “Stabilimento Allevatore, Fornitore Utilizzatore” S.A.F.U., IFO, Roma (4/03/2010). All surgery was performed under sodium pentobarbital anesthesia, and all efforts were made to minimize suffering.

RH30 cells (2×10^6^) stably transfected with either pcDNA3.0\Notch3IC (RH30-Notch3IC) or pcDNA3.0 vector (RH30-Vector) were injected subcutaneously into the posterior legs of nude mice in a cold mixture of PBS\Matrigel (ratio 1∶1). When tumors became palpable, about 15 days after the initial inoculation, their growth was monitored and measured twice weekly by caliper. Tumor volume was calculated with the following formula: tumor volume (mm^3^)  = L×S^2^×π/6 wherein L is the longest and S the shorter diameter and π/6 is a constant to calculate the volume of an ellipsoid. Relative volume was plotted against time in days to determine tumor growth. Representative tumor growth data were obtained from at least 5 mice per experimental group.

In a second set of experiments, animals were sacrificed at specific time points, the tumors were excised and portions were embedded in paraffin and snap-frozen in OCT for immunohistochemical analysis. Additionally, portions were frozen in liquid nitrogen to analyze Notch3IC expression by Western blot. Ten µm sections cut from xenograft blocks were stained with hematoxylin/eosin. Five µm sections were immunostained for Ki67 and HES1 with antibodies and methods as described for primary human RMS samples in Table S2. Counterstaining was carried out with Gill's hematoxylin (Bio-Optica, Milan, Italy). Sections were dehydrated and mounted in non-aqueous mounting medium. Images were acquired through an Eclipse E600 microscope (Nikon, Sesto Fiorentino, Firenze, Italy) at 400X Magnification. Sections were scored using LUCIA software, version 4.81 (Nikon, Sesto Fiorentino, Firenze, Italy) with a Nikon Digital Camera DXM1200F.

### RNA interference

Cells were transfected with 100 nM (final concentration) of double-stranded synthetic 21-mer RNA oligonucleotides (siRNA) using Oligofectamine (Invitrogen, Carlsbad, CA, USA). The most effective JAG1 (NM_000214.2) and DLL1 (NM_005618.3) siRNA, chosen among three different tested Silencer Select siRNAs (Applied biosystems, CA, USA/Ambion, Life Technologies Italia, Monza (MI), Italy) are referenced as s1176 and s26277, respectively. The non-targeting Silencer Select Control N. 1 was the control siRNA (Applied Biosystems, CA, USA/Ambion, Life Technologies Italia, Monza (MI), Italy). siRNA effectiveness was validated by Western blotting and RT-PCR (data not shown).

### Ethics statement

Archival, de-identified formalin-fixed, paraffin-embedded primary RMS and skeletal muscle control tissues were obtained from the Ospedale Pediatrico Bambino Gesù in Roma, University of Padova and Fondazione IRCCS Istituto Tumori of Milano (Italy) after approval of the respective Ethical Committees (EC of Ospedale Pediatrico Bambino Gesù, Roma; EC of the Fondazione IRCCS Istituto Nazionale dei Tumori, Milano; EC of University Hospital of Padova, Padova). We confirm that written informed consent from each donor or parent/guardian when applicable was obtained for use of these samples in research.

### Clinical specimens and immunohistochemistry

Clinicopathological characteristics of the cohort are reported in Table S1. Histopathological features of the tumors were reviewed for the present study by a Pathologist of each Institution (R.B., R.A and P.C) blinded to the results of immunohistochemical analysis. All alveolar tumor samples harbored either PAX3-FOXO or PAX7-FOXO fusion transcripts. Embryonal samples were negative for these gene fusions. Marker expression was evaluated in sections from samples with sufficient available material. Immunohistochemistry was performed with antibodies and immunohistochemistry conditions reported in Table S2. Sections were scored by two independent observers blinded to the identity of the samples and, in rare cases of discrepancy, by an additional third independent observer. Marker positivity was determined on the basis of discrete nuclear staining and a semi-quantitative score of the percentage of positive stained cells/field as the average obtained by scoring at least 5 fields per section. Images were acquired through an Eclipse E600 microscope (Nikon, Sesto Fiorentino, Firenze, Italy) at 400X Magnification. Sections were scored using LUCIA software, version 4.81 (Nikon, Sesto Fiorentino, Firenze, Italy) with a Nikon Digital Camera DXM1200F.

### Statistical analysis

Immunohistochemical expression was analyzed by non-parametric tests. Continuous variables were analyzed by Mann-Whitney U or Kruskal-Wallis test for pairwise or multiple comparisons, respectively. The χ^2^-test or Fisher's exact test was used for comparing categorical variables between groups. Statistical significance was set at a two-tailed P value less than 0.05. All analyses were performed with SPSS 11.5.1 for Windows Package (© SPSS, Inc., 1989–2002 and © LEADTOOLS 1991–2000, LEAD Technologies, Inc., Chicago, NC, USA).

## Results

### Forced expression of a cleaved form of Notch3 (Notch3IC) increases cell proliferation and soft-agar colony formation in vitro of RMS cell lines

**Figure 1:**
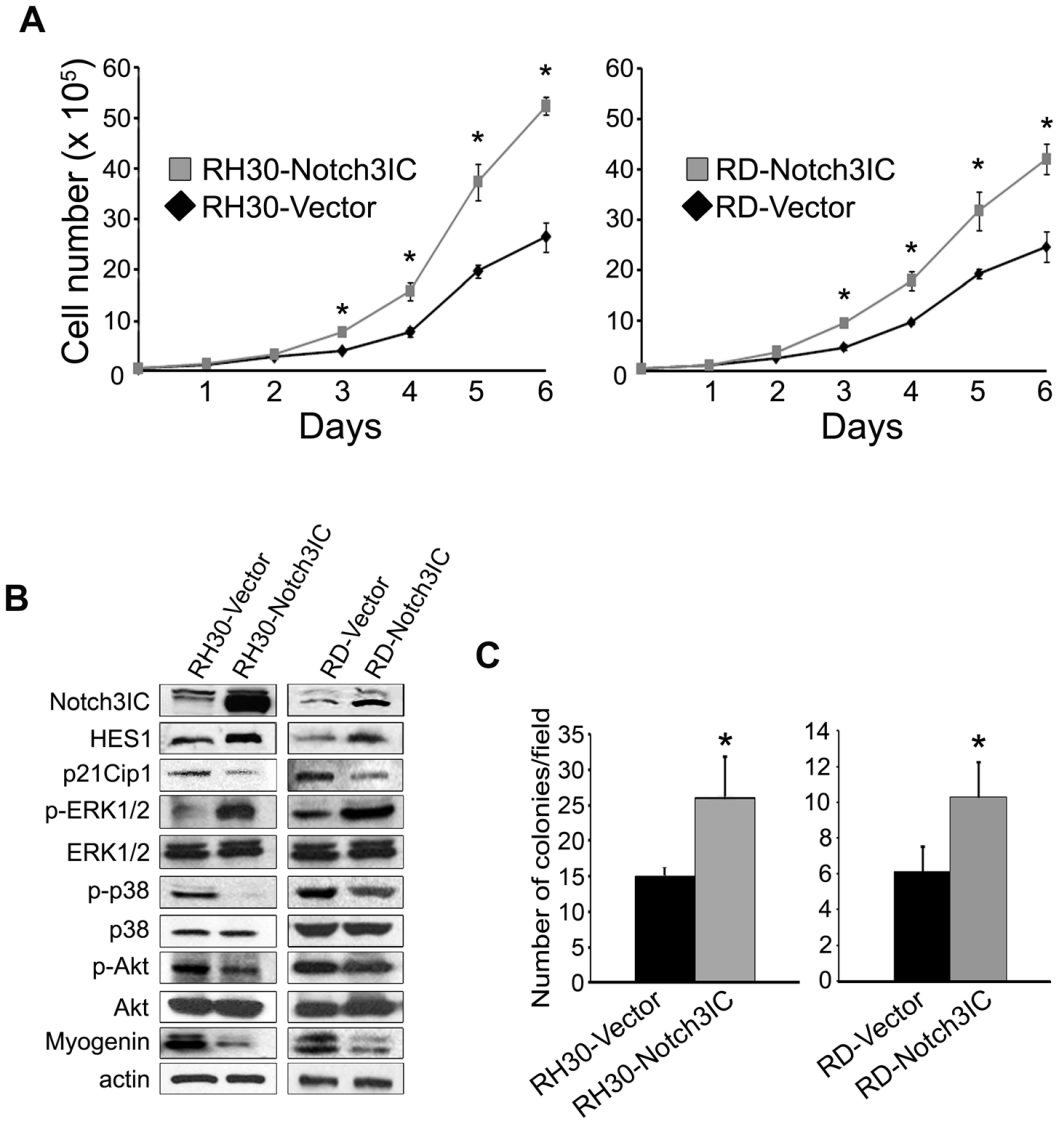
Forced expression of Notch3IC in RH30 and RD RMS cell lines enhances cell proliferation and soft-agar colony formation *in vitro*. A, cell proliferation was assessed on RH30 and RD cells stably transfected with Notch3IC-expressing plasmid (RH30-Notch3IC and RD-Notch3IC) or with an empty control plasmid (RH30-Vector and RD-Vector). Representative of three independent experiments in duplicate (*P<0.05); Bars, SD. B, Western blotting showing the levels of the intracellular active form of Notch3 (Notch3IC), HES1, p21Cip1, phosphorylated ERK1/2 (p-ERK1/2), total ERK1/2 (ERK1/2), phosphorylated Akt (p-Akt), total Akt (Akt), phosphorylated p38MAPK (p-p38), p38MAPK (p38) and Myogenin in RH30 and RD cells stably transfected with either a pcDNA3 expressing Notch3IC (Notch3IC) or an empty pcDNA3 vector as control (Vector). Actin was the loading control. C, 30000 living cells (evidenced by trypan blue exclusion) stably over-expressing Notch3IC (RH30-Notch3IC and RD-Notch3IC) and their control (RH30-Vector and RD-Vector) were seeded on soft-agar in 35-mm Petri dishes and the number of colonies per field were quantified under a light microscopy.

To explore the impact of Notch3 activation on the proliferative potential of RMS cells, we transfected the PAX3-FOXO1-positive alveolar RH30 and PAX3-FOXO1-negative embryonal RD cell lines with a plasmid over-expressing an exogenous cleaved intracellular (active) domain of Notch3, i.e. Notch3IC. Stably Notch3IC-over-expressing RH30 and RD cells showed an increased rate of proliferation, evident as early as 3 days post-seeding. At day 6 post-seeding, Notch3IC cell number had increased by 8.5 fold (RH30 cells) and 7.8 fold (RD cells) compared to 5.2 fold and 4.5 fold for control vector-transfected cells, respectively (Figure 1A). This phenomenon was associated to HES1 protein level up-regulation as compared to empty vector-transfected control cells (Figure 1B). Consistently, both RH30- and RD-Notch3IC cells displayed higher levels of ERK1/2 phosphorylation (Figure 1B). In line with previous results on Notch3 depletion and with the anti-differentiation role of Notch3 in the same cell lines [19], Notch3IC over-expression decreased the levels of both cyclin-dependent kinase inhibitor p21Cip1 and Myogenin along with the activation/phosphorylation of p38 MAPK (mitogen-activated protein kinase) and serine-threonine kinase Akt, all essential for terminal muscle differentiation (Figure 1B). Similar results were obtained with an additional, commercially available PAX3-FOXO1-positive alveolar RMS cell line (RH41) transiently transfected with Notch3IC or control vector (Figure S1A and B).

Of note, Notch3IC over-expression also markedly increased the ability of RH30 and RD cells to form colonies in soft-agar as compared to control vector (Figure 1C).

### Notch3IC increases the *in vivo* tumorigenic potential of RH30 cells

**Figure 2:**
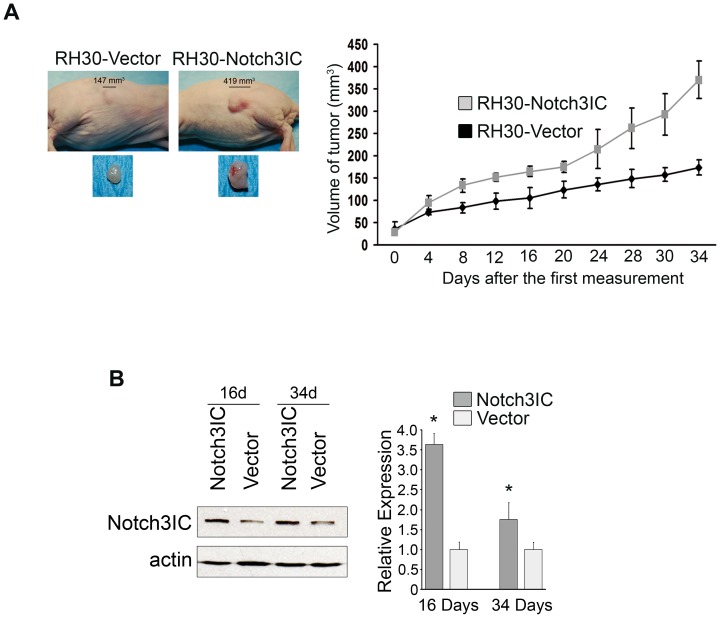
Forced expression of Notch3IC in PAX3-FOXO1 alveolar RH30 cells favors tumor growth *in vivo*. A, nude mice were injected with RH30-Notch3IC or RH30-Vector cells and the volume of tumor xenografts was monitored for 34 days (Right), after which xenografts were surgically removed (Left). Right, mean of tumor volumes (n = 5 mice/group) was calculated and plotted against time in days (RH30-Notch3IC vs RH30-Vector xenograft volumes: P = 0.036 at the 4th day and P = 0.009 for each other time point, Kruskal Wallis test). B, Left, Western blot showing levels of Notch3IC in a pool of 3 xenografts each from mice inoculated with RH30-Notch3IC and RH30-vector cells and excised 16 and 34 days after the first measurement. Actin was the loading control; Right, Histograms depict the densitometric analysis of the western blot shown in the left panel. Columns, means; Bars, SD.

**Figure 3:**
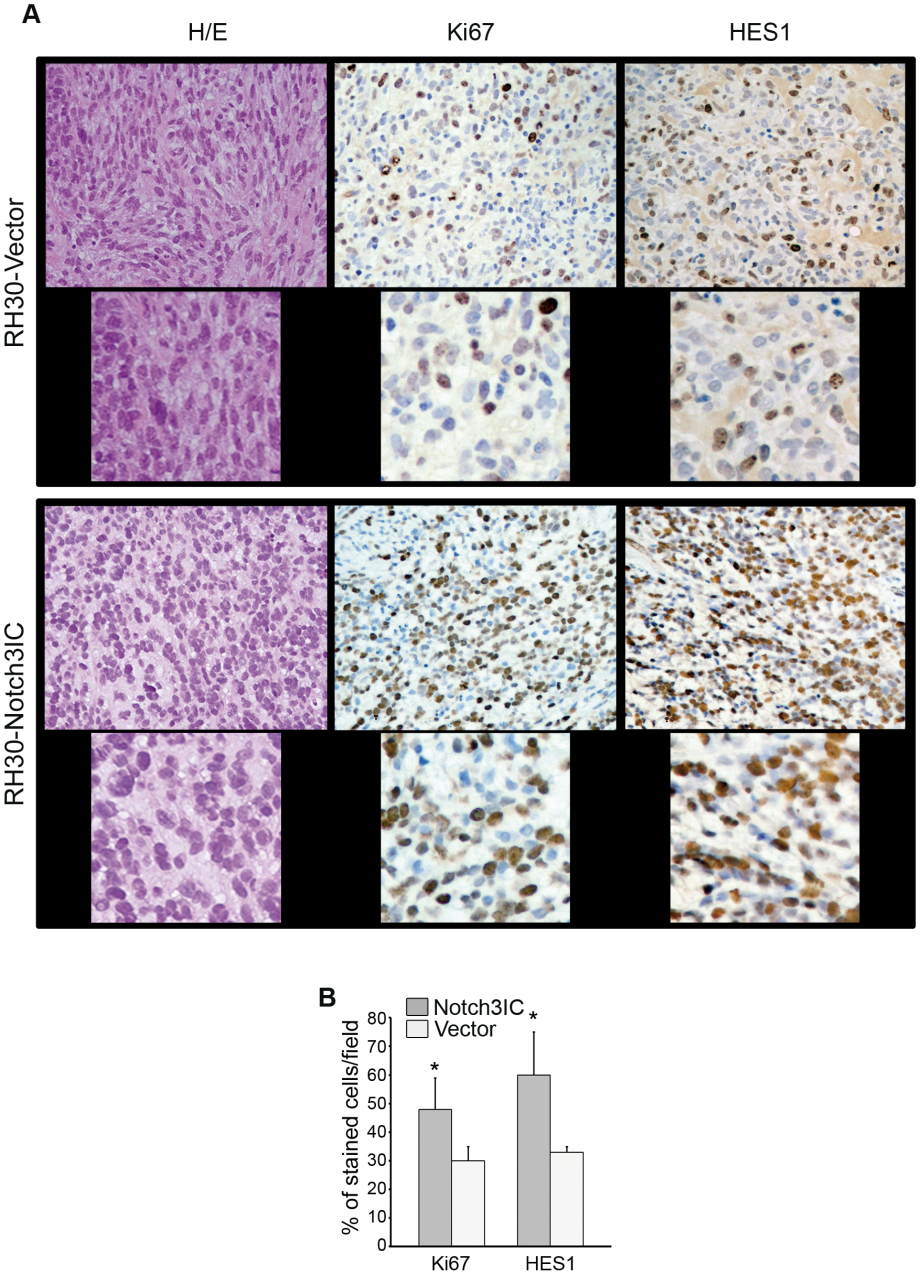
Forced expression of Notch3IC in PAX3-FOXO1 alveolar RH30 cells results in HES1 and Ki67 up-regulation *in vivo*. A, Haematoxylin/eosin staining along with Ki67 and HES1 immunolabeling of 5 µm xenografts serial sections from mice injected with RH30-Notch3IC or RH30-Vector cells and sacrificed between 8 and 12 days after the first tumor volume measurement. Representative of 5 xenografts per condition (Upper: 200X Magnification, Lower: 600X Magnification of selected regions. Brown-orange staining indicates positivity for the marker. B, Histograms depict the percentage of stained (positive) nuclei per field/5 fields for Ki67 and HES1 markers semiquantified under light microscopy. Columns are mean values from 3 mice per group; Bars, SD.

Next, we decided to explore the effect of Notch3IC over-activation on the *in vivo* growth potential of the aggressive PAX3-FOXO1 alveolar RH30 cell line. RH30-Notch3IC or control RH30-Vector cells were subcutaneously inoculated in immunocompromised mice and the growth of xenografted tumors was monitored. A significant difference in tumor volume between the two groups of animals was detected by the 4th day after the first measurement (see Methods), with Notch3IC-expressing cells consistently giving rise to larger tumors (Figure 2A). The sustained over-expression of Notch3IC was verified by Western blotting in tumor xenografts excised at days 16 and 34 (Figure 2B). Consistent with an increased proliferative capability, Ki67 expressing cells were more numerous in xenografts derived from RH30-Notch3IC cells. RH30-Notch3IC cells also expressed higher levels of Notch3 target gene HES1 compared to RH30-Vector cells (Fig 3A and B). Altogether, these results indicate that Notch3 hyper-activation amplifies the proliferative potential of RMS cells in vitro and in vivo.

### Notch3IC over-expression overcomes the anti-proliferative effect of γ-secretase inhibitor DAPT in RMS cells

**Figure 4:**
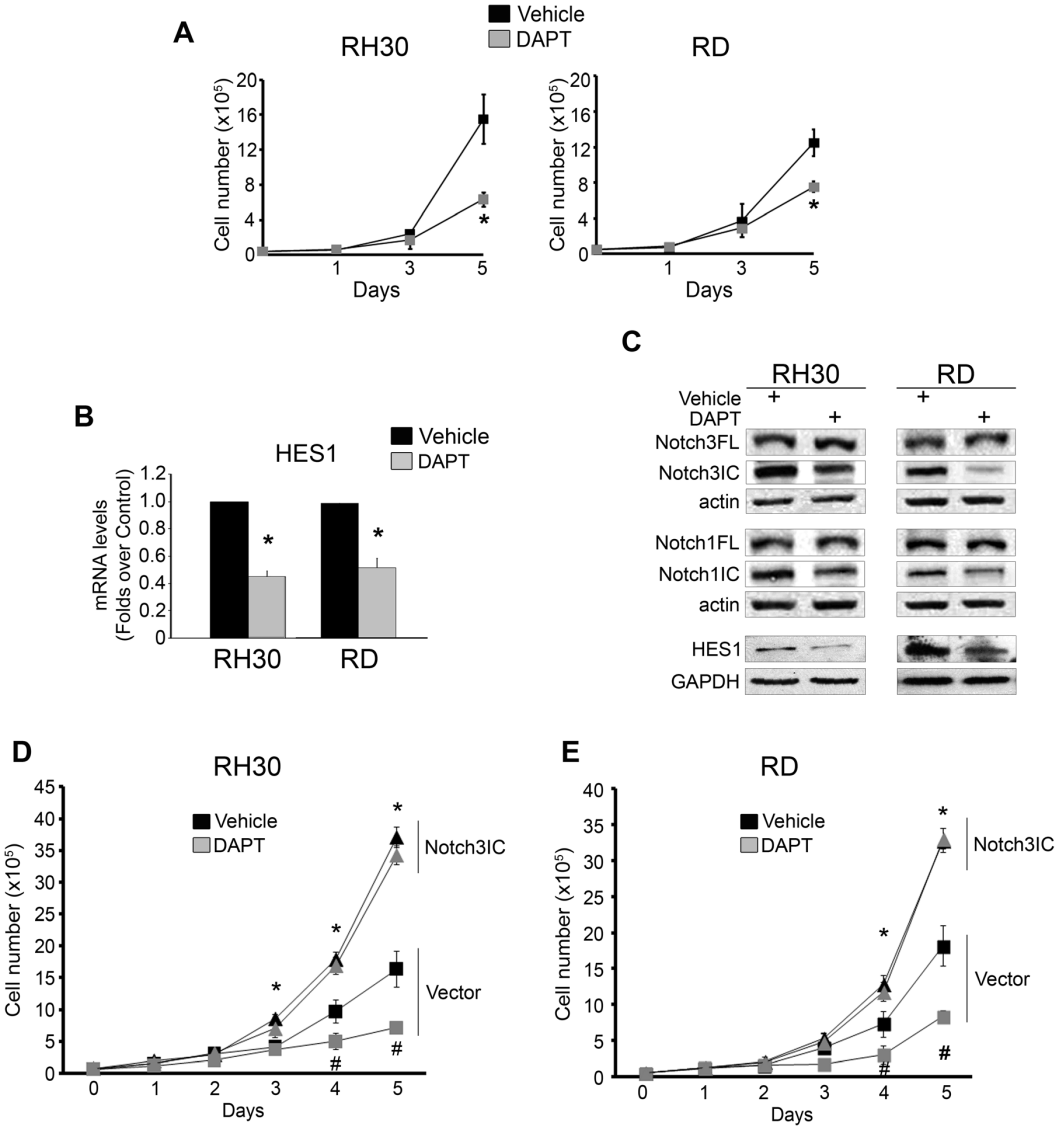
Notch3IC forced expression rescues the anti-proliferative effect of γ-secretase inhibition in both RH30 and RD cell lines. A, cell proliferation was assessed on RH30 and RD cells treated with the γ-secretase inhibitor DAPT (5 µM) or Vehicle (DMSO) and then counted at the reported time points. *P<0.05. B, mRNA levels of HES1 were determined by real time RT-PCR in RH30 and RD cells 72 h after treatment with DAPT (5 µM) or Vehicle (DMSO). Values normalized to actin levels were expressed as fold increase over vehicle-treated cells (1 arbitrary unit). Two independent measurements were done in duplicate. C, Western blotting showing the expression of full length Notch3 (Notch3FL), Notch3IC, Notch1FL, Notch1IC along with that of HES1 72 h after DAPT treatment. Actin and GAPDH were loading controls. D, RH30 and RD cells stably transfected with an empty plasmid (Vector) or with a Notch3IC-expressing plasmid (Notch3IC) were treated with DAPT (5 µM) or Vehicle (DMSO) and then counted at the reported time points. Representative of three independent experiments in duplicate (*P<0.05: Notch3IC vs Vector values; #P<0.05: DAPT vs Vehicle values); Bars, SD.

Inhibitors of γ-secretase (GSIs) non-selectively block the activation of Notch signaling in many cell types [33]. GSIs block the final protelolytic cleavage that releases the Notch intracellular domains (and those of other transmembrane proteins). The anti-proliferative effect of GSIs has been reported for embryonal RMS cell lines [20], [21]. Here, we examined the effect of GSI N-[N-(3,5-Difluoro-phenacetyl)-L-alanyl]-S-phenylglycine t-butyl Ester (DAPT) on the proliferation of RH30 and RD cells cultured in a pro-proliferative medium (i.e., supplemented with 10% FCS). Five days of treatment with 5 µM DAPT decreased proliferation by 60% in RH30 cells compared to vehicle (Figure 4A). A smaller but still significant effect (34%) was seen in RD cells. These findings were associated with significant decrease of HES1 transcripts and protein levels 3 days post-treatment in RH30 and RD cells, respectively, confirming the effectiveness of DAPT in blocking canonical Notch signaling (Figure 4B and C). RH41 cells were also sensitive to DAPT and showed HES1 down-regulation (Figure S2A, B and C).

Similar results were obtained on the RH30 and RD cells with a chemically distinct γ-secretase inhibitor, GSI XII (Figure S3). A marked decrease of the levels of Notch3IC associated to an increase of uncleaved receptor (Notch3FL) was observed after 3 days of DAPT treatment compared to vehicle controls for all the cell lines tested, confirming inhibition of Notch3 cleavage (Figure 4C and Figure S2C). Interestingly, a decrease in Notch1IC accompanied by a modest increase in Notch1FL was observed in RH30 and RH41 cells treated with DAPT, while this effect was more marked in RD cells (Figure 4C and Figure S2C).

To assess whether forced expression of Notch3IC could overcome the anti-proliferative effect of DAPT, we performed a rescue experiment treating Notch3IC cells with the GSI. Treatment with DAPT significantly decreased cell proliferation in empty vector-transfected RMS cells as early as 4 days after treatment (Figure 4D and E, and Figure S2D), as reported for wild-type cells. Conversely, all the Notch3IC-over-expressing RMS cell lines were insensitive to DAPT (Figure 4D and E, and Figure S2D). These findings suggest that forcing Notch3 activation increases RMS cell proliferation and renders cells GSI-resistant, irrespective of their fusion oncoprotein expression or the inhibition of other Notch paralogs.

### The hyper-activation of endogenous Notch3 in RMS cells is ligand-dependent

**Figure 5:**
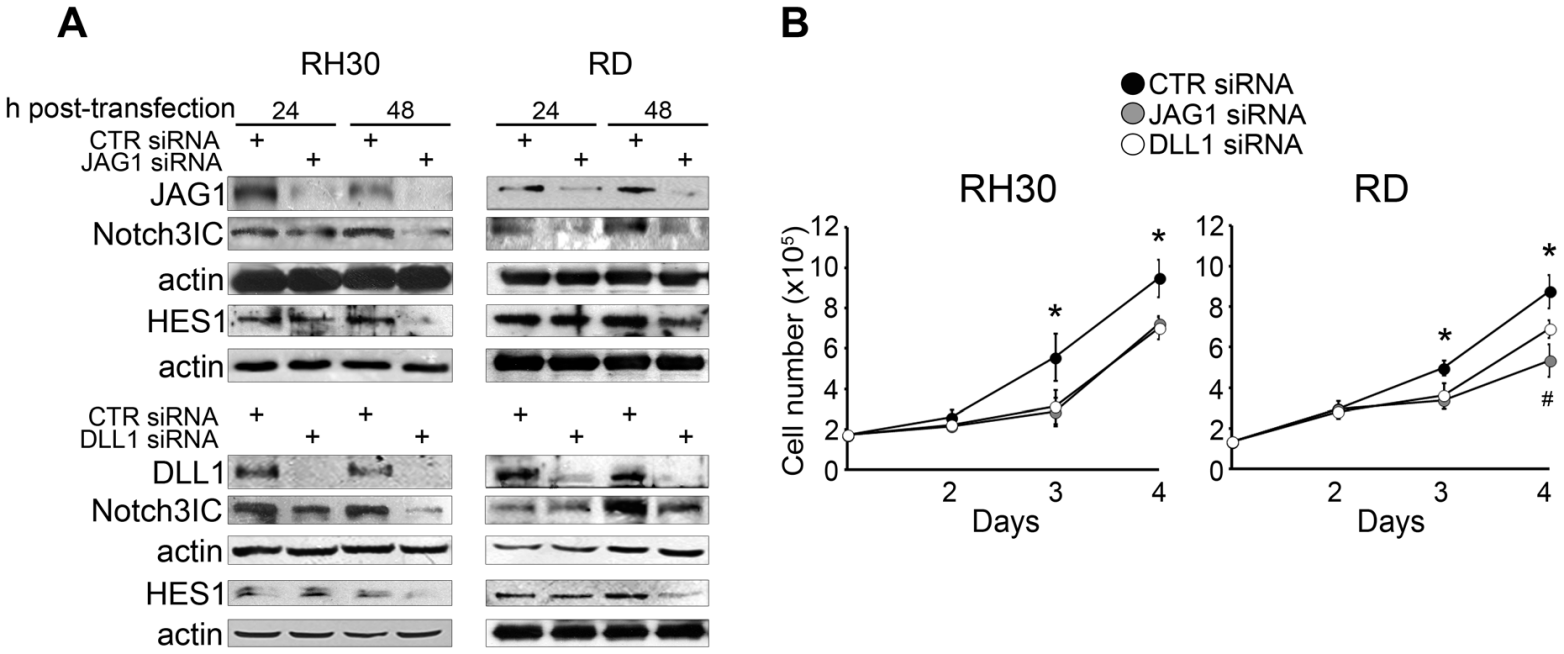
JAG1 and DLL1 down-regulation inhibits Notch3 cleavage and impairs RMS cell proliferation. A, Western blotting (representative of three independent experiments) showing levels of Notch3IC and HES1 in RH30 and RD cells cultured in complete medium (i.e. supplemented with 10% of fetal calf serum) 24 h and 48 h after control (CTR), JAG1 or DLL1 siRNA transfection. Actin was the loading control. B, RH30 and RD cells were treated with control (CTR), JAG1 or DLL1 siRNA and then counted at the reported time points. Data are from three independent experiments in duplicate (*P<0.05: either JAG1 or DLL1 siRNA *vs* CTR siRNA values; #P<0.05: JAG1 siRNA vs DLL1 siRNA values); Bars, SD; Bars, SD.

Hyper-activation of Notch receptors in pediatric leukemias is often, though not always, due to mutations of Notch genes that allow ligand-independent cleavage [28], [29], [30], [31]. This is rarely true for solid cancers, in which very few Notch-activating mutations have been described [34], [35]. We previously showed that Notch3 cleavage is partly dependent on binding to the Serrate/Jagged family Notch canonical ligand JAG1 [19]. Having detected the expression of the Delta-like Notch canonical ligand DLL1 in RMS cells (data not shown), we evaluated whether DLL1 as well contributes to Notch3 activation in these cells. As shown in Figure 5A, a marked down-regulation of the Notch3IC form was detected not only after JAG1 but also after DLL1 knockdown by specific siRNAs in RH30 and RD cells compared to cells transfected with non-targeting control (CTR) siRNAs. RH41 cells behaved similarly (Figure S4A). To elucidate the functional effects of ligand knockdown, we investigated the proliferative potential of siRNAs-treated cells. A significant anti-proliferative response was seen after either JAG1 or DLL1 knockdown in both RH30 and RD cells as early as 3 days after treatment and was maintained up to 4 days (Figure 5B). This effect, though still present 4 days post-treatment, was delayed in RH41 cells (Figure S4B). Therefore, the activation of Notch3 in RMS cells is dependent, at least in part, on canonical ligands.

### Notch3 is activated and HES1 protein levels up-regulated in RMS primary samples

**Figure 6:**
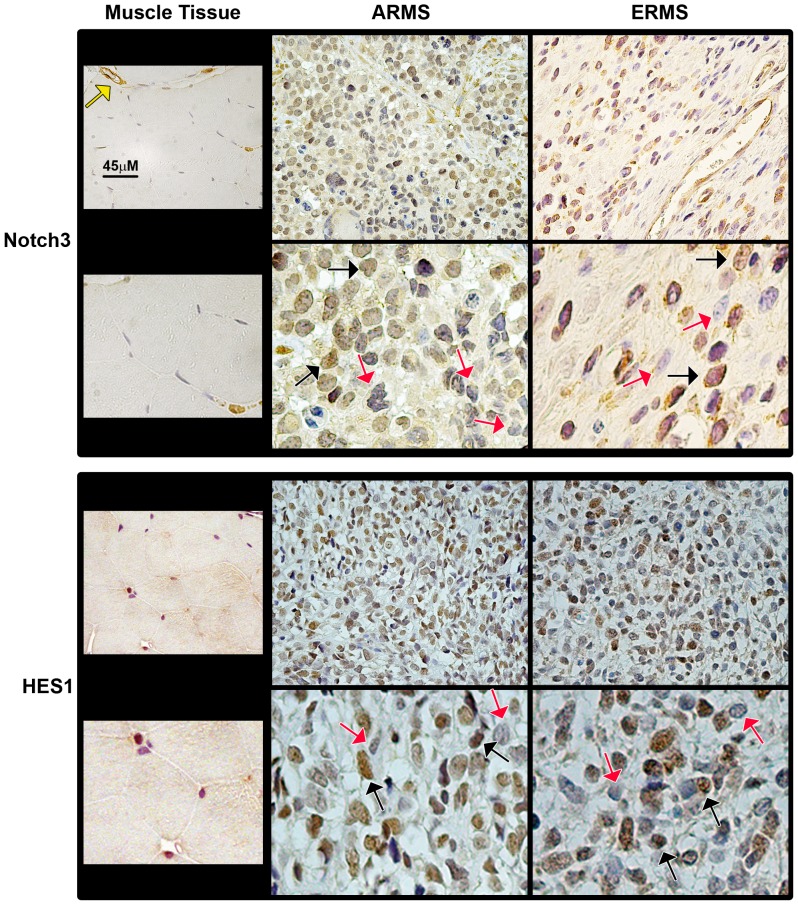
Notch3 and HES1 nuclear protein staining is positive in primary RMS tissues. Representative immunohistochemical staining showing expression of Notch3 and HES1 in alveolar (ARMS; PAX-FOXO1-positive) and embryonal (ERMS) RMS tissue sections (200X Magnification). The lower pictures depict selected regions at higher magnification (600X) for each marker. Black and red arrows indicate nuclei either positive (brown) or negative (blue-gray) for each marker, respectively. Left panels report the immunohistochemistry for each marker in normal skeletal muscle used as control tissue, in which myofibers' nuclei are negative for the two markers. Yellow arrows: vessels with vascular smooth muscle cells positive for Notch3 expression (orange staining).

**Figure 7:**
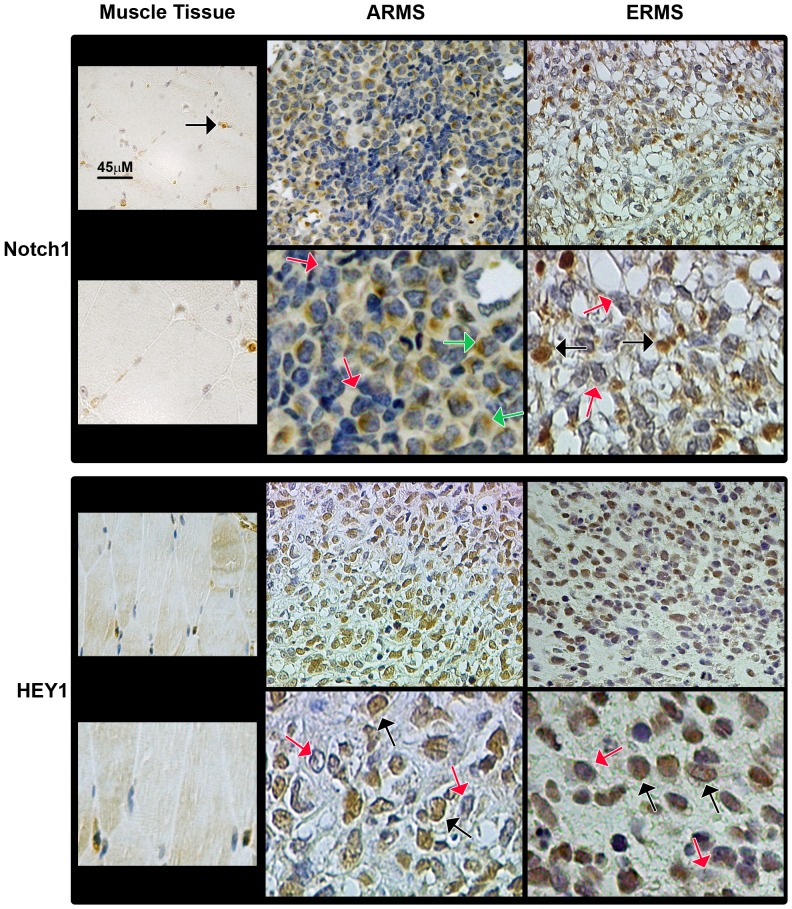
Notch1 and HEY1 nuclear protein staining is positive in primary RMS tissues. Representative immunohistochemical staining showing expression of Notch1 and HEY1 in alveolar (ARMS; PAX-FOXO1-positive) and embryonal (ERMS) RMS tissue sections (200X Magnification). The lower pictures depict selected regions at higher magnification (600X) for each marker. Black and red arrows indicate nuclei either positive (brown) or negative (blue-violet) for each marker, respectively. Green arrows: Notch1 cytoplasmic staining. Left panels report the immunohistochemistry for each marker in normal skeletal muscle used as control tissue, in which myofibers' nuclei are negative for the two markers. A Notch1-positive nucleus of vessel cell was highlighted with black arrow.

**Figure 8:**
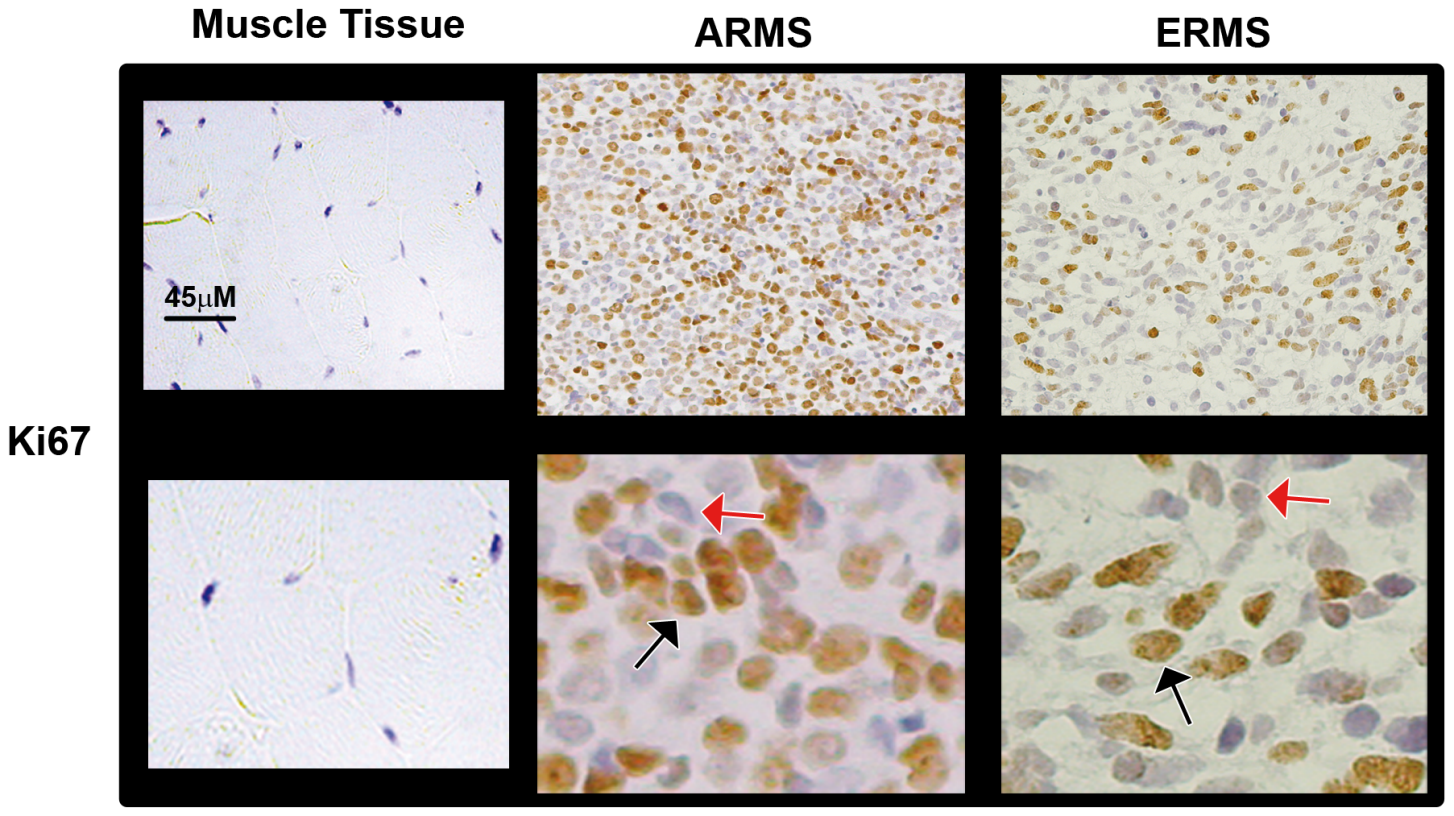
Ki67 staining is positive in primary RMS tissues. Representative immunohistochemical stainings showing expression of Ki67 in alveolar (ARMS; PAX-FOXO1-positive) and embryonal (ERMS) RMS tissue sections (100X Magnification). The lower pictures depict selected regions at higher magnification (600X). Black and red arrows point on nuclei either positive (brown-orange) or negative (blue) for each marker, respectively. Left panels report the immunohistochemistry for Ki67 in normal skeletal muscle used as control tissue, in which myofibers' nuclei are negative for the marker.

The transcript levels of Notch1 and Notch3, together with their respective target genes in RMS, i.e. HEY1 and HES1, in RMS primary samples have been examined by several groups [18], [21], [36]. Therefore, here we evaluated the nuclear expression of the two Notch receptors and of their direct target genes. To this end, we performed immunohistochemical analyses on primary RMS samples. Due to the evidence that fusion-negative alveolar tumors seem to be molecularly and clinically indistinguishable from embryonal ones [37], we examined PAX3-FOXO1- (n = 10) and PAX7-FOXO1-positive (n = 2) alveolar (n = 12 in total) and fusion-negative embryonal (n = 20) RMS samples (Table S1). A well defined nuclear expression, i.e. the presence of the transcriptionally active form of the receptor, was considered to be a marker of Notch receptor activation. We detected higher nuclear expression of Notch3 and HES1 (Figure 6), Notch1 and HEY1 (Figure 7) along with that of Ki67 (Figure 8) in RMS samples compared to control skeletal muscle tissues whose myofibers were negative for all markers (Table 1).

**Table 1:** Immunohistochemical Scoring of Notch Signaling Components in the whole cohort of pediatric patients with rhabdomyosarcomas (RMS) and in alveolar (PAX3-FOXO1, n = 10; PAX7-FOXO1, n = 2) and embryonal (n = 20) RMS subtypes.

Markers	RMS	Alveolar RMS		Embryonal RMS	
**Notch3**	**(n = 31)**	**(n = 12)**		**(n = 19)**	
% of positive nuclei/field	29 (0–76)	30 (5–72)	† ***P*** ** = 0.002**	26 (0–76)	† ***P*** ** = 0.016**
**HES1**	**(n = 32)**	**(n = 12)**		**(n = 20)**	
% of positive nuclei/field	37.5 (12–77)	38 (23–77)	^‡^ ***P*** ** = 0.008**	33.5 (12–62)	
**Notch1**	**(n = 32)**	**(n = 12)**		**(n = 20)**	
% of positive nuclei/field	8.5 (0–44)	4 (0–16)		18 (0–44)	* ***P*** ** = 0.003**
**HEY1**	**(n = 20)**	**(n = 9)**		**(n = 11)**	
% of positive nuclei/field	27.5 (0–70)	8 (0–26)		50 (12–70)	* ***P*** **<0.0001**
**Ki67**	**(n = 32)**	**(n = 12)**		**(n = 20)**	
% of positive nuclei/field	31 (6–60)	43 (23–60)		29 (6–48)	* ***P*** ** = 0.004**

Data are expressed as median values semi-quantified as the percentage of positive nuclei per microscopic field (range in parentheses). Alveolar tumors were confirmed as PAX-FOXO1 fusion positive and Embryonal tumors as PAX-FOXO1 fusion-negative).

*P: Mann-Whitney test for ARMS *vs* ERMS values;

† *P* and ‡*P*: Fisher's exact test for Notch3 *vs* Notch1 and HES1 *vs* HEY1 values within each RMS subtype, respectively.

No significant difference was noticed in the numbers of nuclei expressing either Notch3 or HES1 between PAX-FOXO1 alveolar and embryonal RMS samples (Table 1). Instead, consistent with a previous report despite somewhat lower numbers [21], the fractions of nuclei positive for Notch1 and HEY1 were higher in the embryonal subset. However, in PAX-FOXO1-positive patients, Notch3- and HES1-stained nuclei were significantly more numerous than nuclei positive for Notch1 and HEY1 (Table 1). These results suggest that the Notch3-HES1 axis may be a major Notch-activated signaling pathway in PAX-FOXO1-positive RMS. Moreover, Notch3 and HES1 levels directly correlated with Ki67 in this subgroup (r = 0.676, P = 0.016 and r = 0.644, P = 0.024, respectively).

In summary, our data suggest that Notch3 promotes the proliferation of RMS cells in vitro and in vivo and that a Notch3-HES1 axis may support the proliferation of some pediatric RMS.

## Discussion

We previously demonstrated that Notch3 activation, in part through the expression of its target gene HES1, prevents the *in vitro* differentiation of both embryonal and PAX3-FOXO1-positive alveolar RMS cell lines and sustains the in vivo growth of PAX3-FOXO1 alveolar cells [19]. We showed that Notch3 knockdown is sufficient to reverse these tumorigenic features [19].

Here, we provide evidence that forced over-expression of exogenous Notch3 activated domain, Notch3IC, further stimulates cell proliferation and anchorage-independent growth of both PAX3-FOXO1 alveolar and embryonal RMS cells in vitro, even in medium supplemented with 10% FCS that strongly supports the proliferation of RMS cells. Our observations are consistent with data of Nagao et al. on embryonal RMS RD cells over-expressing an exogenous RBP-Jk protein, suggesting that forcing Notch signaling activation can enhance the intrinsic pro-tumorigenic characteristics of RMS cells [20]. Notch3IC cells expressed higher levels of ERK1/2 phosphorylation and lower levels of p21Cip1 as compared to control vector cells, consistent with stimulation of cell cycle progression. The concomitant down-regulation of Myogenin, Akt and p38 MAPK indicates a concomitant anti-differentiation effect.

Importantly, GSI-mediated inhibition of Notch signaling strongly reduced cell proliferation in both RMS cell subtypes. GSI DAPT markedly affected the cleavage of Notch3 and the expression of its target gene HES1, suggesting endogenously high activity of this Notch paralog in RMS cells. Consistent with this observation, Notch3IC over-expression completely prevented the anti-proliferative effects of GSI in all RMS cell lines, ruling out a cell line- or cell subtype-dependent effect. Even though these results may suggest that γ-secretase inhibition has a Notch3-targeted effect in RMS cells, recent results revealed a similar response when exogenous Notch1IC was expressed in embryonal RD cells [21]. Results from Linardic' group [21] and our data support the hypothesis that hyper-activation of either Notch1 or Notch3 signaling is sufficient to rescue the effects of GSI, suggesting that: 1) these Notch receptors converge on the same proliferative nodal points, at least in embryonal RMS cells and 2) when expressed in sufficient amounts, Notch1IC and Notch3IC can be functionally redundant in these cells. Further studies may elucidate whether this is also true for PAX3-FOXO1 RMS cells.

RH30-Notch3IC cell inoculation in nude mice resulted in the formation of larger tumors compared to vector control cells. Notch3IC expression was maintained in these tumors. Since excessive Notch signaling activity is often toxic for several cell types [38], this result suggests that Notch3 hyper-activation is well tolerated in PAX3-FOXO1-harboring RMS cells and supports tumor growth.

JAG1 and DLL1 knockdown experiments indicate that in the RMS cell lines we evaluated, Notch3 activation is ligand-dependent and both JAG family and DLL family ligands can trigger Notch3 activation. Ligand-independent activation has been described in other tumor cell types [28], [29], [30], [31]. Silencing of either JAG1 or DLL1 mirrored the anti-proliferative effect of GSI, although the effect size was markedly lower. Whether this difference is due to incomplete ligand knockdown or to the fact that both ligand families contribute to Notch3 activation in these cells remains unclear. However, collectively, our data strongly support a pro-proliferative role for Notch3 signaling in RMS cells.

Roma et al. [18] first showed de-regulation of Notch signaling in RMS patients, quantifying transcript levels for several Notch components, including the ones reported in this study. Subsequently, the Linardic group analyzed the nuclear protein expression of Notch1 and its target gene HEY1, as surrogates of Notch1 signaling activation, in primary RMS samples [21]. Here, we performed a similar pilot study to assess Notch3 signaling activation levels. Our findings show that Notch3 is activated and HES1 over-expressed in primary samples from RMS patients, irrespective to their fusion status, compared to normal skeletal muscle tissue. Interestingly, even though the staining fraction was lower in our cohort compared to previous reports [21], Notch1 and HEY1 nuclear expression were significantly higher in embryonal RMS samples. However, in PAX-FOXO1-positive alveolar RMS samples, nuclear levels of Notch3 and HES1 were markedly higher than those of Notch1 and HEY1. This finding suggests that the Notch3-HES1 axis could be a major driver in alveolar RMS bearing PAX3-FOXO1 translocations in vivo. We also noticed that the levels of Notch3 and HES1 were both correlated with proliferation markers in these tumors, suggesting that a hyper-activated Notch3-HES1 axis may contribute to tumor aggressiveness in vivo [3]. However, given that Notch3 hyper-activation showed a similar effect in both tumor cell subtypes, we can argue that the proliferative effects of Notch3IC do not absolutely require expression of the PAX3-FOXO1 fusion oncoprotein. This hypothesis is corroborated by our previous results showing that Notch3 knockdown is detrimental for cells of both RMS subtypes [19], and by data from the Helman group demonstrating that Notch signaling is not among the molecular pathways regulated by PAX3-FOXO1 in RH30 and RH4 RMS cell lines [39]. However, the evidence that Notch3 correlates with Ki67 only in PAX3-FOXO1-positive RMS suggests that simultaneous activation of the Notch3 and PAX3-FOXO1 pathways is a common occurrence in this subset of RMS [37], [40], highlighting once more the difference between the RMS subtypes [41], [42]. In any case, further studies are needed to understand whether and how Notch3 and PAX3-FOXO1 crosstalk or converge on common pathways in RMS.

In conclusion, these results support a role for Notch3 as an amplifier of the proliferative features of RMS cells and suggest that therapeutic targeting of Notch3 may potentiate the effects of conventional therapy in pediatric RMS.

## Supporting Information

Figure S1 **Forced expression of Notch3IC in PAX3-FOXO1-positive RH41 RMS cells enhances cell proliferation** ***in vitro*** **.** **A**, Cell proliferation was assessed on PAX3-FOXO1-positive RH41 cells transiently transfected with either a pcDNA3 plasmid expressing Notch3IC (RH41-Notch3IC) or with an empty pcDNA3 plasmid as control vector (RH41-Vector). Seventy-two hours after transfection polyclonal cell populations were treated with G418 for 1 week and, then, seeded in a 6wells plate, harvested and counted at the reported time points. Representative of two independent experiments in triplicate (*P<0.05); Bars, SD. **B**, RH41 cells were transfected as in (**A**) with either a pcDNA3 expressing Notch3IC (RH41-Notch3IC) or an empty pcDNA3 plasmid as control vector (RH41-Vector) and analyzed 3 days after seeding. Western blotting showing the levels of the intracellular active form of Notch3 (Notch3IC), HES1, p21Cip1, phosphorylated ERK1/2 (p-ERK1/2), total ERK1/2 (ERK1/2), phosphorylated Akt (p-Akt), total Akt (Akt), phosphorylated p38MAPK (p-p38), p38MAPK (p38) and Myogenin. GAPDH was the loading control.
(JPG)

Figure S2 **Notch3IC forced expression rescues the anti-proliferative effect of γ-secretase inhibition in RH41 cells.** **A**, cell proliferation was assessed on RH41 cells treated with the γ-secretase inhibitor DAPT (5 µM) or vehicle (DMSO) and then counted at the reported time points. *P<0.05. **B**, HES1 mRNA levels were determined by real time RT-PCR in RH41 cells 72 h after treatment with DAPT (5 µM) or vehicle (DMSO). Values normalized to actin levels were expressed as fold increase over vehicle-treated cells (1 arbitrary unit). Two independent measurements were done in duplicate. **C**, Western blotting showing the expression of full length Notch3 (Notch3FL), Notch3IC, Notch1FL, Notch1IC along with that of HES1, 72 h after DAPT treatment. Tubulin was the loading control. **D**, RH41 cells were transiently transfected with either a pcDNA3 plasmid expressing Notch3IC (Notch3IC) or with an empty pcDNA3 plasmid as control vector (Vector). Seventy-two hours after transfection polyclonal cell populations were treated with G418 for 1 week and, then, seeded in a 6wells plate, treated with DAPT (5 µM) or vehicle (DMSO) and then harvested and counted at the reported time points. Representative of two independent experiments in triplicate (* *P*<0.05); Bars, SD.
(JPG)

Figure S3 **Inhibition of γ-secretase with GSI XII impairs cell proliferation of RMS cells.** **A**, cell proliferation was assessed on RH30 and RD cells treated with the γ-secretase inhibitor GSI XII (5 µM) or vehicle (DMSO) and then counted at the reported time points. *P<0.05. **B**, HES1 mRNA levels were determined by real time RT-PCR in RH30 and RD cells 72 h after treatment with GSI XII (5 µM) or vehicle (DMSO). Values normalized to actin levels were expressed as fold increase over vehicle-treated cells (1 arbitrary unit). Two independent measurements were done in duplicate.
(JPG)

Figure S4 **JAG1 and DLL1 down-regulation inhibits Notch3 cleavage and impairs RH41 cell proliferation.** **A**, Western blotting showing levels of Notch3IC and HES1 in RH41 cell line cultured in complete medium (i.e. supplemented with 10% of fetal calf serum) 48 h and 72 h after control (CTR), JAG1 or DLL1 siRNA transfection. Actin was the loading control. Representative of three independent experiments. **B**, RH30 and RD cells were treated with control (CTR), JAG1 or DLL1 siRNA and then counted at the reported time points. Representative of three independent experiments in duplicate (* *P*<0.05: either JAG1 or DLL1 siRNA *vs* CTR siRNA values); Bars, SD.
(JPG)

Table S1 **Clinical and histopathological features of pediatric patients with alveolar (PAX3-FOXO1-positive n = 10; PAX7-FOXO1-positive n = 2) and embryonal (n = 20) rhabdomyosarcoma (RMS).**
(DOC)

Table S2 **Antibodies and Conditions for Immunohistochemistry on Primary Pediatric Rhabdomyosarcoma Samples.**
(DOC)
