# Supplementary figures and images for: Hyper-Activation of Notch3 Amplifies the Proliferative Potential of Rhabdomyosarcoma Cells
Source: PLoS One. 2014 May 5;9(5):e96238. doi: 10.1371/journal.pone.0096238 (PMC4010457; doi:10.1371/journal.pone.0096238)

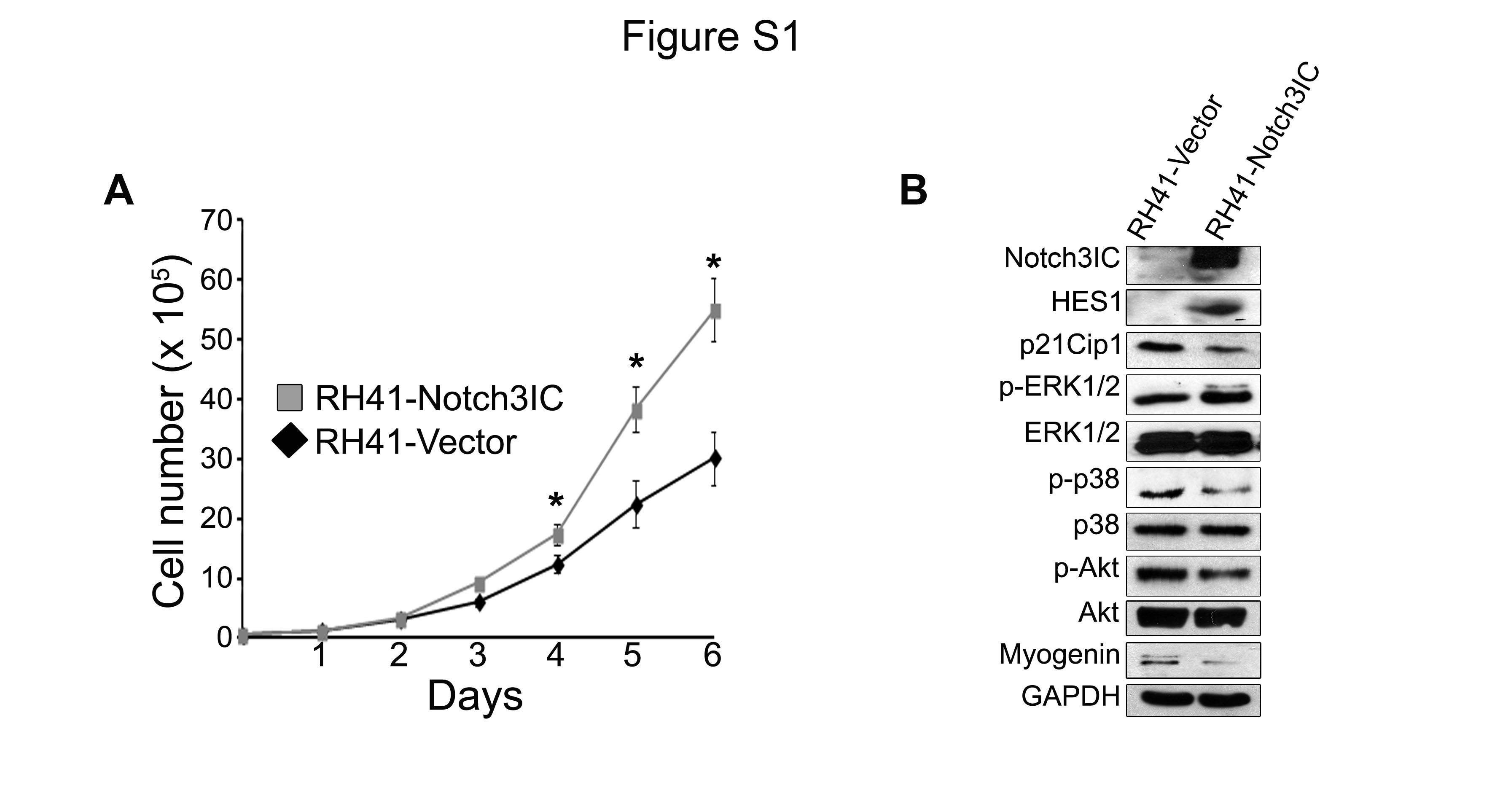

Supplement: Figure S1 — Forced expression of Notch3IC in PAX3-FOXO1-positive RH41 RMS cells enhances cell proliferation in vitro . A, Cell proliferation was assessed on PAX3-FOXO1-positive RH41 cells transiently transfected with either a pcDNA3 plasmid expressing Notch3IC (RH41-Notch3IC) or with an empty pcDNA3 plasmid as control vector (RH41-Vector). Seventy-two hours after transfection polyclonal cell populations were treated with G418 for 1 week and, then, seeded in a 6wells plate, harvested and counted at the reported time points. Representative of two independent experiments in triplicate (*P<0.05); Bars, SD. B, RH41 cells were transfected as in (A) with either a pcDNA3 expressing Notch3IC (RH41-Notch3IC) or an empty pcDNA3 plasmid as control vector (RH41-Vector) and analyzed 3 days after seeding. Western blotting showing the levels of the intracellular active form of Notch3 (Notch3IC), HES1, p21Cip1, phosphorylated ERK1/2 (p-ERK1/2), total ERK1/2 (ERK1/2), phosphorylated Akt (p-Akt), total Akt (Akt), phosphorylated p38MAPK (p-p38), p38MAPK (p38) and Myogenin. GAPDH was the loading control. (JPG) [file pone.0096238.s001.jpg]

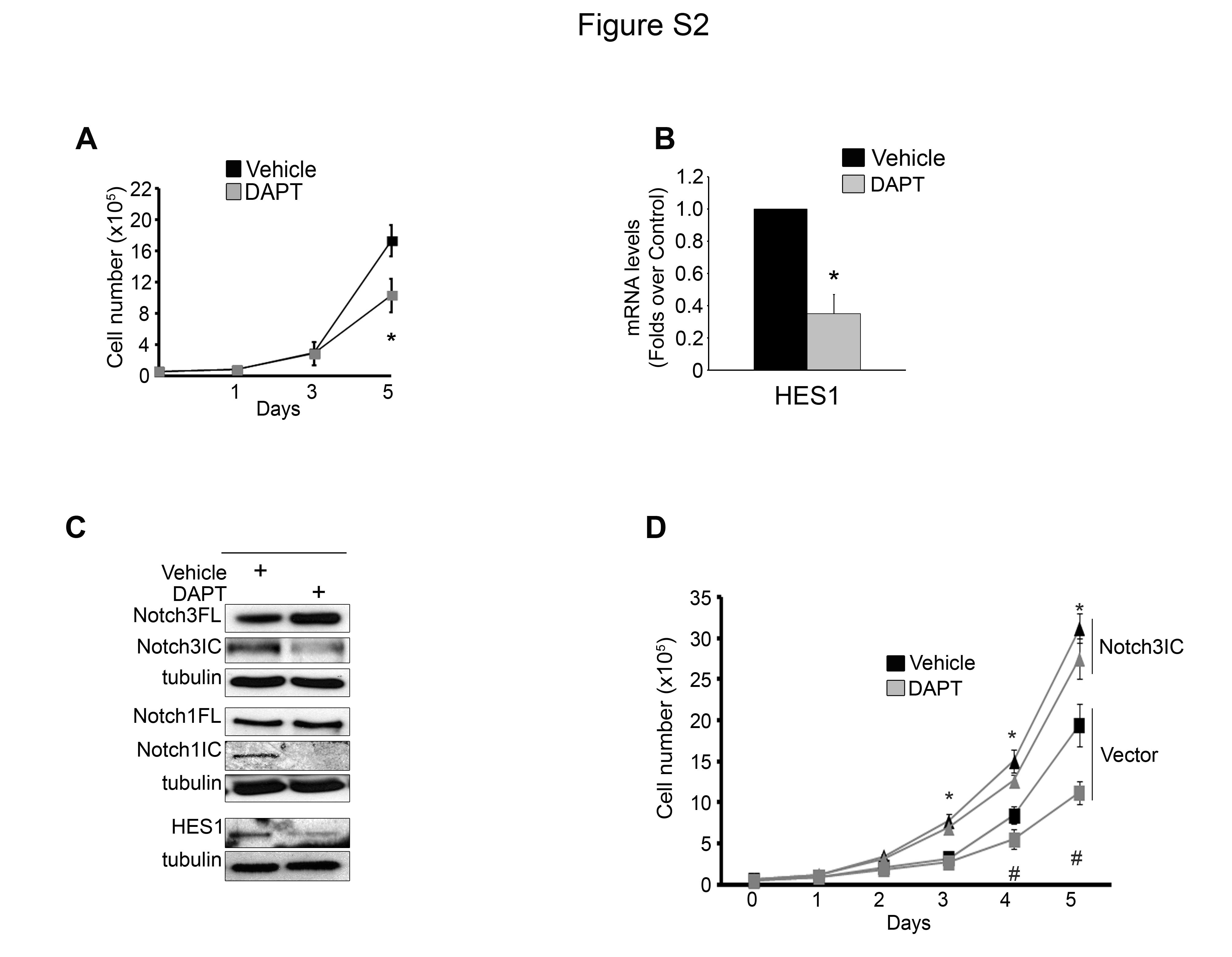

Supplement: Figure S2 — Notch3IC forced expression rescues the anti-proliferative effect of γ-secretase inhibition in RH41 cells. A, cell proliferation was assessed on RH41 cells treated with the γ-secretase inhibitor DAPT (5 µM) or vehicle (DMSO) and then counted at the reported time points. *P<0.05. B, HES1 mRNA levels were determined by real time RT-PCR in RH41 cells 72 h after treatment with DAPT (5 µM) or vehicle (DMSO). Values normalized to actin levels were expressed as fold increase over vehicle-treated cells (1 arbitrary unit). Two independent measurements were done in duplicate. C, Western blotting showing the expression of full length Notch3 (Notch3FL), Notch3IC, Notch1FL, Notch1IC along with that of HES1, 72 h after DAPT treatment. Tubulin was the loading control. D, RH41 cells were transiently transfected with either a pcDNA3 plasmid expressing Notch3IC (Notch3IC) or with an empty pcDNA3 plasmid as control vector (Vector). Seventy-two hours after transfection polyclonal cell populations were treated with G418 for 1 week and, then, seeded in a 6wells plate, treated with DAPT (5 µM) or vehicle (DMSO) and then harvested and counted at the reported time points. Representative of two independent experiments in triplicate (* P<0.05); Bars, SD. (JPG) [file pone.0096238.s002.jpg]

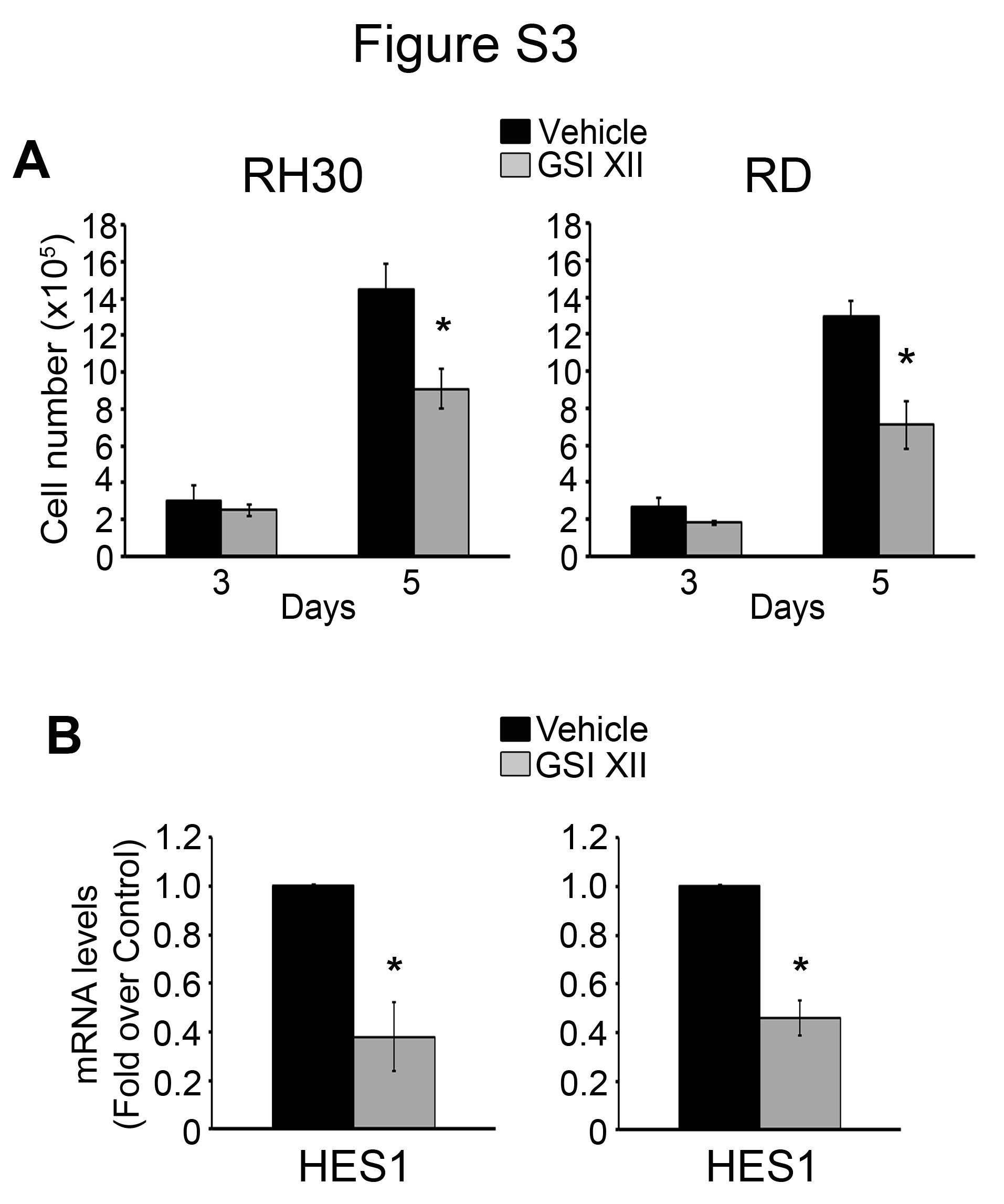

Supplement: Figure S3 — Inhibition of γ-secretase with GSI XII impairs cell proliferation of RMS cells. A, cell proliferation was assessed on RH30 and RD cells treated with the γ-secretase inhibitor GSI XII (5 µM) or vehicle (DMSO) and then counted at the reported time points. *P<0.05. B, HES1 mRNA levels were determined by real time RT-PCR in RH30 and RD cells 72 h after treatment with GSI XII (5 µM) or vehicle (DMSO). Values normalized to actin levels were expressed as fold increase over vehicle-treated cells (1 arbitrary unit). Two independent measurements were done in duplicate. (JPG) [file pone.0096238.s003.jpg]

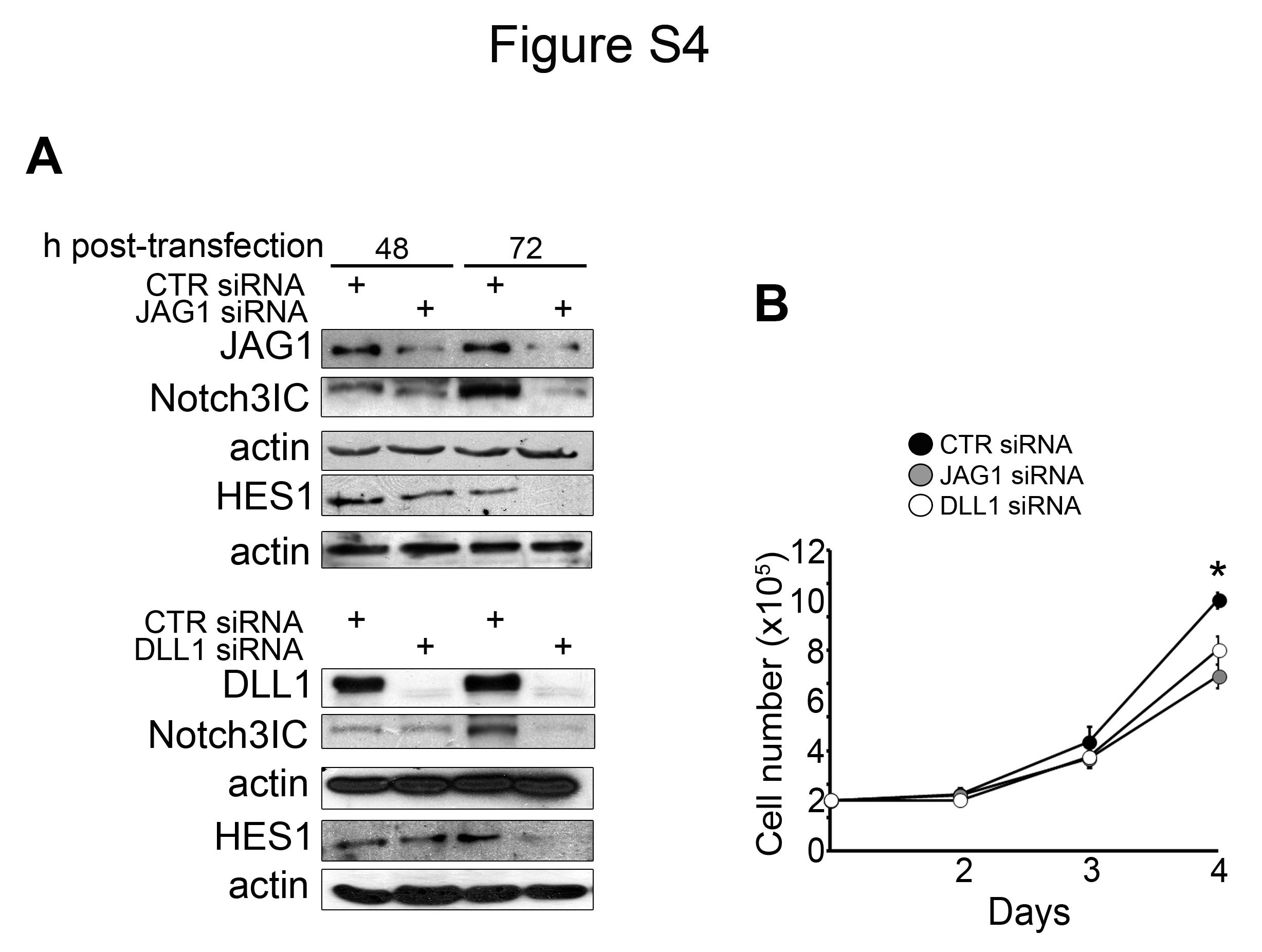

Supplement: Figure S4 — JAG1 and DLL1 down-regulation inhibits Notch3 cleavage and impairs RH41 cell proliferation. A, Western blotting showing levels of Notch3IC and HES1 in RH41 cell line cultured in complete medium (i.e. supplemented with 10% of fetal calf serum) 48 h and 72 h after control (CTR), JAG1 or DLL1 siRNA transfection. Actin was the loading control. Representative of three independent experiments. B, RH30 and RD cells were treated with control (CTR), JAG1 or DLL1 siRNA and then counted at the reported time points. Representative of three independent experiments in duplicate (* P<0.05: either JAG1 or DLL1 siRNA vs CTR siRNA values); Bars, SD. (JPG) [file pone.0096238.s004.jpg]
