# Supplementary material for: Hyper-Activation of Notch3 Amplifies the Proliferative Potential of Rhabdomyosarcoma Cells
Source: PLoS One. 2014 May 5;9(5):e96238. doi: 10.1371/journal.pone.0096238 (PMC4010457; doi:10.1371/journal.pone.0096238)
Supplement: Table S1 — Clinical and histopathological features of pediatric patients with alveolar (PAX3-FOXO1-positive n = 10; PAX7-FOXO1-positive n = 2) and embryonal (n = 20) rhabdomyosarcoma (RMS). (DOC) [file pone.0096238.s005.doc]

**Table S1.** Clinical and histopathological features of pediatric patients with alveolar (PAX3-FOXO1-positive n=10; PAX7-FOXO1-positive n=2) and embryonal (n=20) rhabdomyosarcoma (RMS)

|  | **alveolar RMS**  **n (%)** | **embryonal RMS**  **n (%)** |
| --- | --- | --- |
| Sex |  |  |
| Male | 4 (33) | 12 (60) |
| Female | 8 (67) | 8 (40) |
| Age (years) |  |  |
| < 10 | 9 (75) | 12 (60) |
| ≥ 10 | 3 (25) | 8 (40) |
| Localisation |  |  |
| Orbit-Genitourinary tract-Head and Neck**^$^** | 5 (42) | 10 (50) |
| Cranial paramenigeal-Extremity-Other**^$$^** | 7 (58) | 10 (50) |
| Tumor volume |  |  |
| < 5 cm | 5 (42) | 7 (35) |
| ≥ 5 cm | 7 (58) | 13 (65) |
| IRS stage |  |  |
| I | 1 (8) | 5 (25) |
| II | 0 (0) | 2 (10) |
| III | 7 (58) | 10 (50) |
| IV | 4 (33) | 3 (15) |
| Metastasis |  |  |
| No | 8 (67) | 18 (90) |
| Yes | 4 (33) | 2 (10) |
| Recurrence |  |  |
| No | 4 (33) | 15 (75) |
| Yes | 8 (67) | 5 (25) |
| Outcome |  |  |
| Alive | 7 (58) | 13 (65) |
| DOD | 5 (42) | 7 (35) |

Abbreviations: DOD, dead of disease; IRS, Intergroup Rhabdomyosarcoma Study Group staging system; **^$^**Favorable and **^$$^**Unfavorable tumor localization.
