# Supplementary material for: Hyper-Activation of Notch3 Amplifies the Proliferative Potential of Rhabdomyosarcoma Cells
Source: PLoS One. 2014 May 5;9(5):e96238. doi: 10.1371/journal.pone.0096238 (PMC4010457; doi:10.1371/journal.pone.0096238)
Supplement: Table S2 — Antibodies and Conditions for Immunohistochemistry on Primary Pediatric Rhabdomyosarcoma Samples. (DOC) [file pone.0096238.s006.doc]

# Table S2. Antibodies and Conditions for Immunohistochemistry on Primary Pediatric Rhabdomyosarcoma Samples

| Antibody | DonorSpecies | Recognized human domain (aa=amino acids) | Source | **Clone** | **Dilution** | **Antigen Retrieval** | Primary antibodies incubation time | Secondary antibodies |
| --- | --- | --- | --- | --- | --- | --- | --- | --- |
| Notch1 | Rat | aa 2219-2377 | DSHB, University of Iowa, Iowa City, IA | bTAN 20 | 1:100 | EDTA (pH8)* | 4°C over-night | UAPBA**†** |
| Notch3 | Rabbit | aa 2306-2321 | Orbigen, Inc.  San Diego, CA | PAB-10683 | 1:100 | Citrate (pH6)**^‡^** | 4°C over-night | Biotinilated link* |
| HES1 | Rabbit | aa 163-194 | Santa Cruz Biotechnology, Inc, Santa Cruz, CA | H-140 | 1:50 | Citrate (pH6)**^‡^** | 4°C over-night | Biotinilated link* |
| HEY1 | Rabbit | Region between residues 207-261 | Abcam, Cambridge, UK | ab22614 | 1:100 | EDTA (pH8)* | 4°C over-night | Biotinilated link* |
| Ki-67 | Mouse | A portion of  1086 base pair | Novocastra Laboratories Ltd, Newcastle upon Tyne, UK | MM1 | 1:200 | Citrate (pH6)**^‡^** | 1h RT | Biotinilated link* |

Abbreviations: DSHB, Developmental Studies Hybridoma Bank; UAPBA, Ultrateck anti polyvalent biotin antibody; RT, room temperature.

*DAKO, Carpintera, CA; **^‡^**UCS Diagnostic, Roma, IT; **†**Scytek Laboratories, Logan, UT.
